# Supplementary material for: Integrative epigenomics in Sjögren´s syndrome reveals novel pathways and a strong interaction between the HLA, autoantibodies and the interferon signature
Source: Sci Rep. 2021 Dec 2;11:23292. doi: 10.1038/s41598-021-01324-0 (PMC8640069; doi:10.1038/s41598-021-01324-0)
Supplement: Supplementary file 1 — Supplementary Information. [file 41598_2021_1324_MOESM1_ESM.docx]

***Supplementary Note1*** *contains a list of clinical centers involved in the PRECISESADS clinical Consortium*

***Supplementary Note2*** *contains a list of investigators involved in the PRECISESADS Flow Cytometry Study Group*
