# Supplementary material for: Integrative epigenomics in Sjögren´s syndrome reveals novel pathways and a strong interaction between the HLA, autoantibodies and the interferon signature
Source: Sci Rep. 2021 Dec 2;11:23292. doi: 10.1038/s41598-021-01324-0 (PMC8640069; doi:10.1038/s41598-021-01324-0)
Supplement: Supplementary file 3 — Supplementary Note 2 [file 41598_2021_1324_MOESM3_ESM.docx]

| **** PRECISESADS Flow Cytometry Study Group:**  Christophe Jamin^1^, Concepción Marañón^2^, Lucas Le Lann^1^, Quentin Simon^1^, Bénédicte Rouvière^1^, Nieves Varela Hernández ^2^, Brian Muchmore^2^, Aleksandra Dufour^3^, Carlo Chizzolini^3^, Jonathan Cremer^4^, Ellen De Langhe^5^, Nuria Barbarroja^6^, Chary Lopez-Pedrera^6^, Velia Gerl^7^, Laleh Khodadadi^7^, Anne Buttgereit^8^, Zuzanna Makowska^8^, Aurélie De Groof^9^, Julie Ducreux^9^, Elena Trombetta^10^, Tianlu Li^11^, Damiana Alvarez-Errico^11^, Torsten Witte^12^, Katja Kniesch^12^, Esmeralda Neves^13^, Sambasiva Rao^14^ and Jacques-Olivier Pers^1^  1 U1227, Université de Brest, Inserm, Labex IGO, CHU de Brest, Brest, France.  2 GENYO, Centre for Genomics and Oncological Research Pfizer, University of Granada, Andalusian Regional Government, PTS GRANADA, Granada, Spain.  3 Immunology & Allergy, University Hospital and School of Medicine, Geneva, Switzerland  4 Laboratory of Clinical Immunology, Department of Microbiology and Immunology, KU Leuven, Leuven, Belgium.  5 Division of Rheumatology, University Hospitals Leuven and Skeletal Biology and Engineering Research Center, KU Leuven, Leuven, Belgium.  6 IMIBIC/Reina Sofia Hospital/ University of Cordoba, Cordoba, Spain.  7 Department of Rheumatology and Clinical Immunology, Charité University Hospital, Berlin, Germany.  8 Bayer Pharma AG, Berlin, Germany.  9 Pôle de Pathologies Rhumatismales Inflammatoires et Systémiques, Institut de Recherche Expérimentale et Clinique, Université catholique de Louvain, Brussels, Belgium.  10 Laboratorio di Analisi Chimico Cliniche e Microbiologia - Servizio di Citofluorimetria, Fondazione IRCCS Ca' Granda Ospedale Maggiore Policlinico di Milano, Milano, Italy.  11 Chromatin and Disease Group, Bellvitge Biomedical Research Institute (IDIBELL), Barcelona, Spain  12 Klinik für Immunologie und Rheumatologie, Medical University Hannover, Hannover, Germany.  13 Serviço de Imunologia EX-CICAP, Centro Hospitalar e Universitário do Porto, Porto, Portugal  14 Sanofi Genzyme, Framingham, MA, USA |
| --- |
|  |
