# Supplementary material for: Integrative epigenomics in Sjögren´s syndrome reveals novel pathways and a strong interaction between the HLA, autoantibodies and the interferon signature
Source: Sci Rep. 2021 Dec 2;11:23292. doi: 10.1038/s41598-021-01324-0 (PMC8640069; doi:10.1038/s41598-021-01324-0)
Supplement: Supplementary file 4 — Supplementary Figure 1 [file 41598_2021_1324_MOESM4_ESM.pdf]

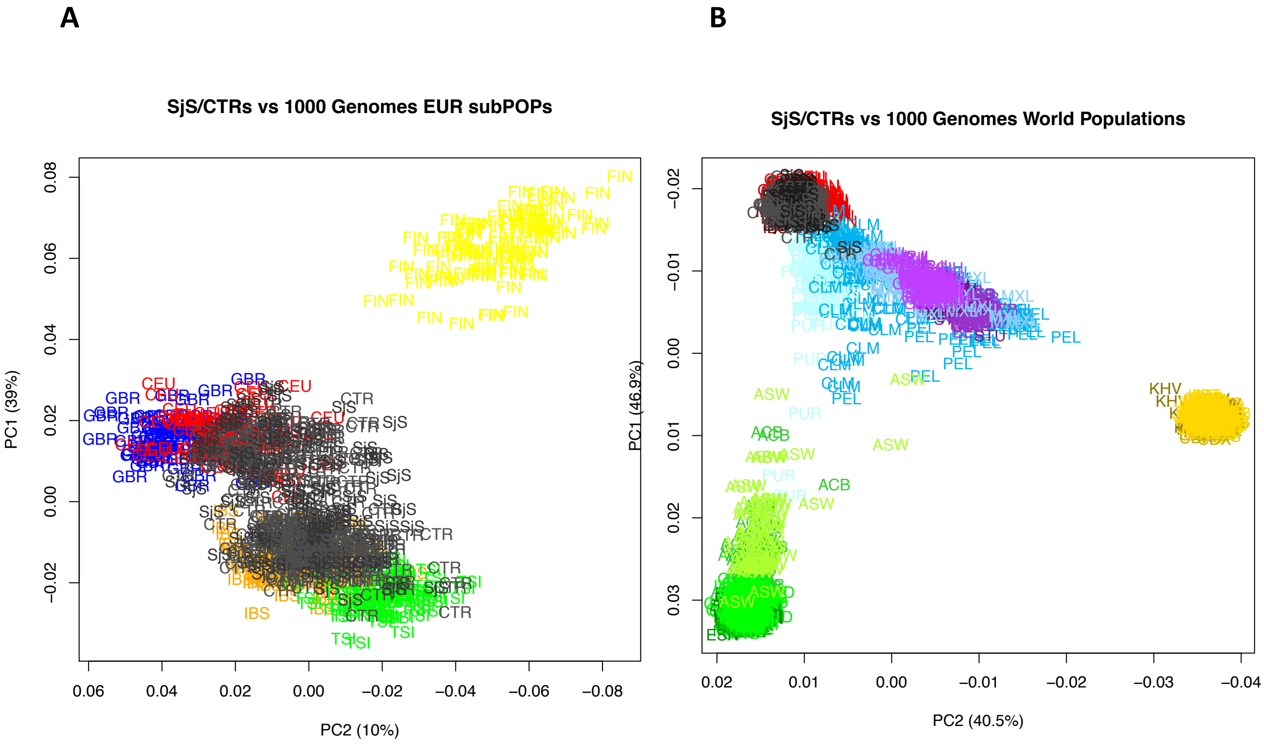

Supplementary Figure 1. Genetic structure of SjS and CTRL samples included in this study. Principal component (PC) analyses of SS and CTRL (gray color). For PC calculation 2516 Ancestry Informative Markers (AIMS) specific for European population were used. A. The two first genetic principal components of SjS and CTRL samples are represented with those from the 1000 G European samples. B. The first two principal components for SjS and CTRL samples are represented with worldwide populations. The first two principal components can explain genetic differences among samples due to population structure of European populations. The first two PCs, PC1 and PC2, explain 87% of the explained variation.

AFR populations [ACB: African Caribbean, ASW: African Ancestry SW, ESN: Esan, GWD: Gambian Mandinka, LWK: Luhya, MSL: Mende and YRI: Yoruba], green colours; AMR populations [CLM: Colombian, MXL: Mexican Ancestry, PEL: Peruvian and PUR: Puerto Rican], blue colours; EAS populations [CDX: Dai Chinese, CHB: Han Chinese, CHS: Southern Han Chinese, JPT: Japanese and KHV: Kinh Vietnamese], yellow colours; EUR populations [CEU: CEPH, FIN: Finnish, GBR: British, IBS: Iberian and TSI: Toscani], red colours; SAS populations [BEB: Bengali, GIH: Gujarati, ITU: Telugu, PJL: Punjabi and STU: Tamil], purple colours.
